# A Greek Pediatric Word Recognition Test by Picture Identification

**DOI:** 10.3390/brainsci13121643

**Published:** 2023-11-27

**Authors:** Nikolaos Trimmis, Konstantina Chatzi, Vasiliki Grammatsoulia, Foteini Feida, Konstantinos Mourtzouchos, Angelos Papadopoulos, Panagiotis Plotas

**Affiliations:** 1 Department of Speech and Language Therapy, University of Patras, 26504 Patras, Greece; nicktrimmis@upatras.gr (N.T.); kwnstantinaxatzh1994@gmail.com (K.C.); vasiliki.gram@gmail.com (V.G.); logotherapycenter@gmail.com (F.F.); angelospapadopoulos@gmail.com (A.P.); 2 Department of Pediatric Otorhinolaryngology, Karamandaneio Children’s Hospital of Patras, 26331 Patras, Greece; 3 Laboratory Primary Health Care, School of Health Rehabilitation Sciences, University of Patras, 26504 Patras, Greece

**Keywords:** word recognition score, WRS, children, picture identification task, speech audiometry

## Abstract

(1) Background: The study aimed to construct a clinically valuable closet-set WRS test with a picture identification task for young Greek-speaking children. (2) Methods: The test material was meticulously designed based on specific criteria. To determine which parts of speech are used more frequently by preschool children, a spontaneous speech sample (250 words per child) was acquired from three hundred children aged 3 to 6 years (M = 4.56, SD = 0.90). The study involved the development and application of two phonemically balanced 50-word lists suitable for young children, as well as the creation of picture representations for each response set. All testing was accomplished in an audiometric booth that exceeded the audiometric rooms’ ambient noise level standards. The speech signal was routed from a laptop computer to a GSI 61 audiometer, and all test items were delivered from the audiometer to the subject. (3) Results: The results indicated that materials for a WRS test for young children are developed with high face validity and are applicable for children as young as three years old. The test satisfies the essential components needed for a WRS test. It consists of two phonemically balanced 50-word lists with low-redundancy bisyllabic words, with each list containing 227 phonemes. (5) Conclusions: This novel closed-set WRS test presents a valuable tool for assessing speech perception skills in young Greek-speaking children. The test results have various applications, including diagnosis, research, and (re)habilitation.

## 1. Introduction

Hearing is crucial for the development of communication skills. Even mild hearing loss can compromise speech, language, psychosocial behavior, and other developmental milestones [1]. A comprehensive audiology evaluation (CAE) is necessary if uncertainty about a child’s hearing status exists. Speech audiometry is a vital part of any CAE as it provides information of greater value than pure-tone audiometry alone. It can be used throughout the auditory system to determine speech-processing abilities [2]. Two common speech audiometry procedures are the Speech-Recognition-Threshold (SRT) and the Word-Recognition-Score (WRS). The SRT measures the threshold of speech and is the lowest intensity level at which a person can correctly recognize 50% of presented speech stimuli [3,4]. The primary objective of WRS testing is to ascertain the estimated suprathreshold threshold above which an individual may accurately comprehend and reproduce a series of words. The resulting score is the proportion of words in a given list that are accurately detected at levels over the threshold [4,5]. In other words, the WRS measures speech understanding at a comfortable hearing level. Behavioral assessment of children will frequently begin with speech audiometry that has revealed hearing abnormalities, or their absence, in young children who were unable or unwilling to take pure-tone tests. This approach has resulted in substantial savings of time in initiating remedial procedures [6]. Some children will not respond to pure tones, and it appears that they can not hear. Many of these children can be conditioned to respond to speech stimuli. A child will often point to a picture or object. 

Speech recognition thresholds (SRT) can frequently be obtained with picture identification by age 2. Word recognition score (WRS) test can be accomplished in the same way and is one of the most frequently used measures for speech audiometry [5,7]. Over the years, researchers have developed several WRS instruments for preschoolers as part of the hearing validation process. However, there is no generally accepted standard test for the speech assessment of young children because speech perception is an abstract construct. Therefore, it is not easy to measure in an absolute sense [8]. The most popular of these measures include the Northwestern University Children’s Perception of Speech Test [9], the Word Intelligibility by Picture Identification Test [10], the Pediatric Speech Intelligibility Test [11], and the Minimal Pairs Test [12]. 

The assessment of WRS in young children presents a challenge to clinicians [13]. To increase reliability, the speech material of the test must be within the child’s receptive vocabulary, and the response mode must be age-appropriate [14,15]. An open-set response mode means the patient must compare the stimulus item to all possible candidate words in lexical memory, whereas a closed-set response mode means the patient is provided with a choice of several possible response alternatives. In addition, it is necessary to have speech audiometry materials available in each individual’s native language for reliable results. 

The majority of the early evaluations of speech intelligibility, as outlined by Miller (1946), utilized open-set tests that focused on the recognition of words or syllables. Nevertheless, many researchers decided to use closed-set speech intelligibility tests due to their expediency and simplicity in terms of administration and scoring [16]. The main concept of the closed-set multiple-choice intelligibility tests is that the word recognition process remains consistent, irrespective of the test’s response style. According to speech and hearing scientists, the only difference between open-set and closed-set tasks was the occurrence of chance performance [16]. In open-set tests, the chance performance is represented by 1 divided by the size of the mental lexicon (L), while in closed-set tests, it is represented by 1 divided by the number of response possibilities offered by the experimenter (N). The closed-set testing format has maintained its popularity in clinical settings due to its efficient and expedient administration and scoring procedures [16]. Closed-set tests have demonstrated their reliability even when conducted with limited trials [16]. Closed-set and open-set tasks differ in terms of information processing needs. Specifically, the level of competition between alternative responses is a fundamental distinction between these two types of tasks. In closed-set tests, the scope of competition among alternatives is constrained to the response set selected by the investigator. In the context of open-set tests for spoken word recognition, it is essential to acknowledge the presence of lexical rivalry that occurs over the entirety of the mental lexicon [16]. At this point, it is crucial to acknowledge that closed-set WRS testing has distinct advantages for implementation compared to open-set WRS testing [17].

Today, while several open-set speech audiometry tests are currently available in Greek for adults and school-aged children, there is still no valid speech test available with a picture-pointing task for pediatric evaluation, especially for children younger than six. Given the above limitations in Greek speech audiometry testing, our goal in this study was to construct a clinically valuable closet-set WRS test with a picture identification task for young Greek-speaking children. More specifically, our primary aims were to evaluate the word familiarity of list items, ensure the phonemic balance of the lists, produce digital recordings of the stimuli, select digitally illustrated pictures for each response set, and conduct a preliminary investigation of list equivalence.

## 2. Materials and Methods

**Figure 1:**
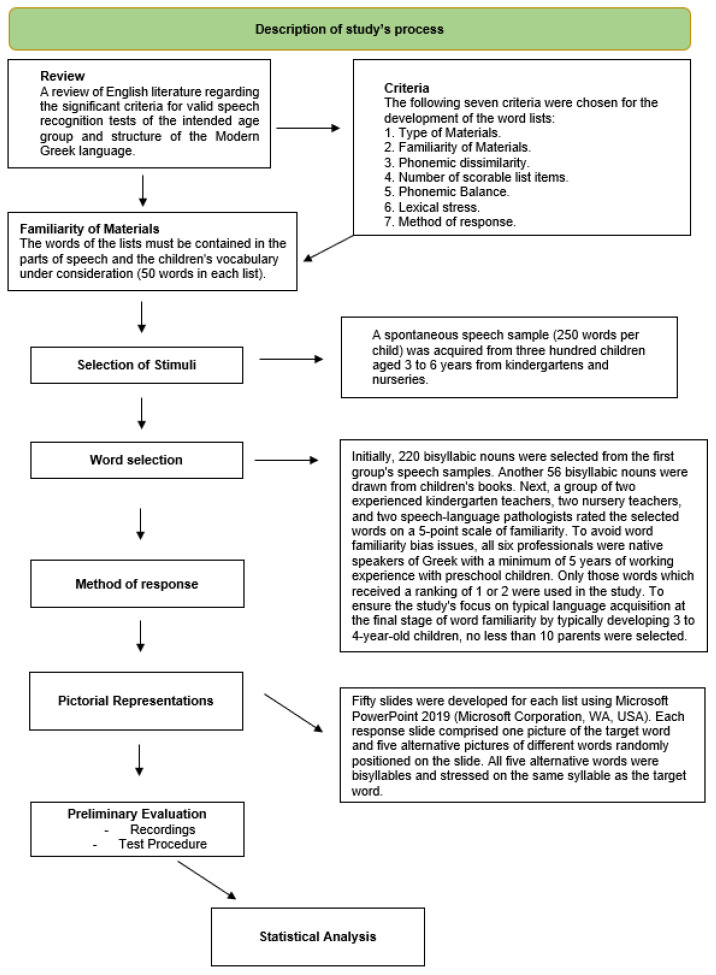
Flow diagram of the study process.

A review of English literature regarding the significant criteria for valid speech recognition tests of the intended age group and structure of the Modern Greek language has guided us to develop a set of word lists specifically for use in preschool children’s WRS measurements. The process of the study is presented in the Figure 1 flow diagram. The following seven criteria were chosen for the development of the word lists:

1. Type of Materials.
2. Familiarity of Materials.
3. Phonemic dissimilarity.
4. Number of scorable list items.
5. Phonemic Balance.
6. Lexical stress.
7. Method of response.

The criteria for five of the above, namely type of materials, phonemic dissimilarity, number of scorable list items, phonemic balance, and lexical stress, were used in previous research [4,18]. The remaining two criteria are discussed below. The institutional review board approved the present study. To avoid methodological biases, standardized procedures were consistent across participants and conditions. 

### 2.1. Familiarity of Materials

The possibilities for constructing speech audiometry materials for young children are indeed more restricted than with older children and adults due to lower stages of language abilities development. Phonemic and linguistic requirements usually have to be adjusted, and more emphasis is placed on the familiarity and simplicity of the test words. The familiarity of a given word enhances the word’s intelligibility [19]. Research has shown that highly familiar word lists offer a more precise indicator of an individual’s continuous discourse perception than less familiar word lists [20].

The selection of speech materials for young children should be within the children’s speech and language competence [2,13]. The words on the lists must be contained in the parts of speech and the children’s vocabulary under consideration. Words fall into various categories called “parts of speech.” For example, “verbs” and “nouns” are some words; “pronouns” and “adjectives” are others. Each of these categories of words represents a part of speech. 

### 2.2. Selection of Stimuli

Parts of speech. To determine which parts of speech are used more frequently by preschool children, a spontaneous speech sample (250 words per child) was acquired from three hundred children aged 3 to 6 years (M = 4.56, SD = 0.90) from kindergartens and nurseries in the cities of Athens and Patras. All children were monolingual speakers of Greek and presented no history of speech and language problems or other developmental disorders. The children were divided into three age groups. The first group corresponded to the age of 3 to 4 years old, the second 4 to 5 years old, and the third 5 to 6 years old. Each group consisted of 100 children (50 boys and 50 girls). The speech samples were elicited through conversational strategies and narratives to produce the most complex language [21]. Each child’s sample was recorded individually for later analysis. Table 1 presents the frequency of occurrence of all parts of Modern Greek speech for the 300 children.

Preschoolers use more nouns followed by verbs in their production of spontaneous speech. There was no statistically significant difference between boys and girls for the nouns (*t* = 0.16, df = 298, *p* > 0.05) and verbs (*t* = 0.66, df = 298, *p* > 0.05). Comparison between the groups revealed that nouns are the most frequently used part of speech in all three groups of participants. The lower the group’s age, the more nouns appeared in spontaneous speech. Specifically, the 3–4-year-old children used 24.25% of nouns, the 4–5-year-old children used 22.59%, and the 5–6-year-old group used 21.10%. A study [22] investigated vocabulary composition in Greek preschool children. Common nouns were the largest category among the fifty most frequent words. Recently, a grammatical content analysis of hard-of-hearing children’s speech in Greece aged 6–14 revealed that nouns have the highest frequency [23].

### 2.3. Word Selection 

The receptive vocabulary size of young children increases rapidly. At age 3, children recognize about 1000 words, and by age 5, they recognize at least 10,000 words [24]. Therefore, since the present study is intended to design a speech test for children of vocabulary level of age 3 through 6 years, the stimulus words must be primarily selected from the subset of word usage in the first group.

However, children’s receptive vocabulary generally exceeds their expressive vocabulary [25]. Thus, children’s books of this age range were also used to select simple bisyllabic nouns that could be adequately represented pictorially.

Initially, 220 bisyllabic nouns were selected from the first group’s speech samples. Another 56 bisyllabic nouns were drawn from children’s books. 

Next, a group of two experienced kindergarten teachers, two nursery teachers, and two speech-language pathologists (six female; average age = 41.24 years; Standard Deviation = 5.77) rated the selected words on a 5-point scale of familiarity (1 = extremely familiar, 2 = very familiar, 3 = somewhat familiar, 4 = infrequently used, and 5 = rarely used). To avoid word familiarity bias issues, all six professionals were native speakers of Greek with a minimum of 5 years of working experience with preschool children. Only those words which received a ranking of 1 or 2 were used in the study. To ensure the study’s focus on typical language acquisition at the final stage of word familiarity by typically developing 3 to 4-year-old children, no less than 10 parents were selected with the following inclusion and exclusion criteria:

The Inclusion criteria included: Female biological parents of children between the ages of 3 and 4 years old, native Greek speaker, graduate of Greek high school, regular interactions with their children, and no medical or developmental concerns that may impact language development. 

Exclusion criteria: Parents who did not satisfy all the above criteria were excluded from the study.

To further enhance familiarity, the 214 words that were rated with 1 or 2 were judged as either familiar or unfamiliar by ten parents (ten female; average age = 32.80 years; Standard Deviation = 3.71) who had in the first group, children with normal hearing bilaterally. The final 192 words rated as familiar were used to construct the test lists.

### 2.4. Method of Response

**Figure 2:**
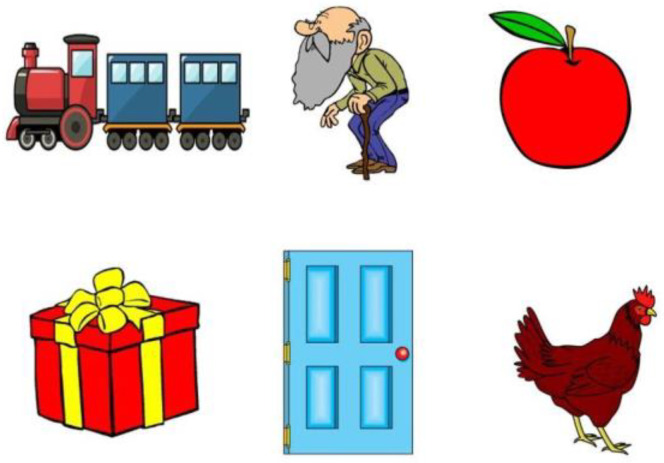
A sample response slide employed in the Picture Identification Task. All six pictures represent bisyllabic words with stress on the first syllable. The image is the property of the author.

The response format used to elicit accurate responses is a crucial issue to consider when designing WRS tests for young children. The type of response required must reflect the child’s ability to hear, and this must not be contaminated by other factors [19]. Some children are too shy to speak in the test room environment and will not provide suitable responses to an open-set test format, where the patient repeats the word that they hear [14]. Also, an oral response is inappropriate for children with limited speech production skills, such as articulatory problems, because the tester cannot determine whether such a response is due to faulty hearing or faulty articulation. Thus, the response format must be age-appropriate and not require speech. This criterion is met with a picture identification pointing response to a set of pictures, a closed-response set. Children’s speech perception can be assessed using picture-pointing tasks when their receptive language skills are 2.6 years of age or greater [2]. Fifty slides were developed for each list using Microsoft PowerPoint 2019 (Microsoft Corporation, Redmond, WA, USA). Each response slide comprised one picture of the target word and five alternative pictures of different words randomly positioned on the slide. All five alternative words were bisyllables and stressed on the same syllable as the target word. American Speech Hearing Association (ASHA) [3] recommended that if pointing responses are employed with picture cards, an excessively high number of items may be distracting and increase response time, and a deficient number of items increases the probability of chance performance. An example of a response plate is presented in Figure 2.

### 2.5. Pictorial Representations

Items selected were simple bisyllabic words that could be easily represented pictorially. The following visual clarity criteria were adopted:

- Pictures with simple, recognizable images.
- Pictures with vibrant colors that are visually appealing to young children.
- Pictures with a strong contrast between the foreground and background.
- Pictures that are age-appropriate for preschoolers.
- Consistent visual style throughout the selection of pictures.
- Pictures do not contain any inappropriate content.

The picture selection was a lengthy process requiring scanning many online sites that provided free stock pictures. Several pictures were selected for each target word. Next, the same group of two experienced kindergarten teachers, two nursery teachers, and two speech-language pathologists working with young children were consulted as experts. The expert group was asked to rate each item for its visual clarity (easily recognizable by young children) on a 3-point scale (1 = high clarity, 2 = average clarity, 3 = low clarity). Only the pictures which received a ranking of 1 were used in the study.

### 2.6. Preliminary Evaluation

#### 2.6.1. Subjects

The individuals who participated in this study were selected from kindergartens and nurseries. They included 50 children ranging in age from 3 to 6 years (25 boys and 25 girls; Mean = 4.68 years; Standard Deviation = 0.80). All participants met the following criteria:

1. Monolingual speakers of Greek.
2. No known history of auditory dysfunction, speech or language delay, or other cognitive disorders.
3. Not currently receiving speech or language therapy.
4. Hearing within normal limits bilaterally. Pure tone thresholds of ≤15 Decibels Hearing Level (dBHL) at all octave frequencies ranging from 250 Hertz (Hz) to 8000 Hz.
5. None of the children were on medication or had known illness on the day of testing.
6. No history of vision problems. All children passed a screening test as measured by a Snellen children’s color blindness chart.

#### 2.6.2. Recordings

Speech audiometry testing can be performed with monitored live voice or recorded material. However, the use of recorded material standardizes the test procedure. Auditory stimuli were recorded in a 40A series audiometric booth (IAC Acoustics, Winchester, UK) by a young adult female native speaker of Modern Greek. The speaker was instructed to maintain clarity, pace, and effort to produce each target word at least three times. The words were digitized with a professional unidirectional microphone at a 44.100 kilohertz (kHz) sampling frequency and 16-bit resolution. Each word was equalized to the average root mean square (RMS) amplitude. Words were presented with a 5-s interval. A 1000-Hz calibration tone of 30 s duration was synthesized and equalized to the average RMS level of the 100 test words. Two experienced judges (one audiologist and one speech pathologist) rated the repetitions of each word for the perceived quality of production, and the best production of each word was selected.

#### 2.6.3. Test Procedure

Before data collection, each participant’s parent signed an informed consent form approved by the Departmental Committee of TEI Western Greece (currently the University of Patras). All testing was accomplished in the same 40A series audiometric booth that exceeded the audiometric rooms’ ambient noise level standards. The speech signal was routed from a laptop computer to a GSI 61 audiometer (Grason-Stadler, Eden Prairie, MN, USA). All test items were delivered from the audiometer to the subject via supra-aural TDH-49 headphones (Telephonics, Farming Dale, NY, USA). Although there were difficulties with younger children maintaining consistent attention with pre-recorded stimuli, all participants could perform the test. Each subject participated in more than one session to reduce performance variability due to the child’s fatigue.

Verbal instructions to point to a picture and motivation were used for each child. A test assistant in the test room, seated next to the child and in visual contact with the examiner, maintained the child’s interest in the task, informed the examiner about the child’s response, and presented a new response slide on the screen after the child made a selection.

Each list was presented monaurally (right ear) at ten consecutive intensity levels, ranging from 0 to 45 dBHL, starting at 0 dBHL and ascending in 5 dBHL steps. Subjects responded to each item by pointing to one of six pictures of the response slide on a laptop computer screen in front of the child. None of the participants was familiarized with the test materials prior to testing. The words within each list and the order of presentation of the lists were randomized for each subject to decrease nonauditory factors such as memory effects. 

### 2.7. Statistical Analysis 

Statistical analysis was performed using the Statistical Package for the Social Sciences (SPSS) version 23.0 software (IBM Corp., Armonk, NY, USA). The normality assumption for all variables was addressed by the Kolmogorov–Smirnov test and Shapiro–Wilk test. An independent *t*-test was conducted to examine the equivalence of the two lists. To determine if there was a significant interaction between different factors (list, gender, and age) on the WRSs (dependent variable), a general linear model repeated measures analysis, consisting of WRSs at different decibel hearing levels (dBHL = 10 levels: OdBHL, 5dBHL, 1OdBHL, 15dBHL, 20dBHL, 25dBHL, 3OdBHL, 35dBHL, 4OdBHL, 45dBHL) as the within-subject variable and list (two levels: list 1 and list 2), gender (two levels: male and female) and age (three levels: 3–4 years, 4–5 years, and 5–6 years) as the between-subject variables, was used. A *p*-value of <0.05 was considered to be statistically significant.

## 3. Results

The limited number of suitable words that could be used with this population and the adoption of the criteria by the authors restricted the possible choices of constructing several word lists. Due to these limitations, developing more than two 50-word lists was impossible. Several 50-word lists are needed for WRS testing to minimize training and memory effects. For example, an audiologist might need to determine WRSs at different intensity levels. Therefore, a different 50-word list should be presented at each decibel hearing level. Table 2 presents the phonemic balance information for the two preschool lists. The lists were modified numerous times to satisfy better the criteria for constructing the final version of the test. 

**Figure 3:**
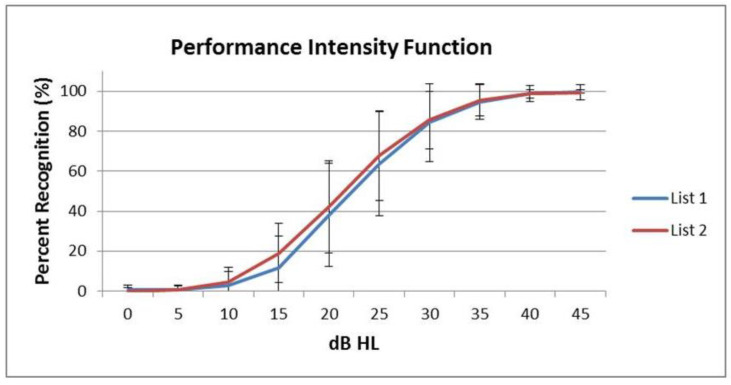
Monaural (right ear) PI functions of 50 normal-hearing children and standard deviations of the two preschool bisyllabic lists. The image is the property of the author.

Each new list consists of 50 bisyllabic nouns (Table 3). Data from the presentation of the two lists at the different intensity levels were used to construct the performance intensity (PI) functions, which represent the relationship between word recognition accuracy and the intensity (volume) level of the presented stimuli. Figure 3 presents the PI functions for the two preschool bisyllabic lists’ mean scores and standard deviations.

Mean WRSs increased with an average slope per decibel of 4.79% in the first list and 4.42% in the second list in the rapidly rising portion of the function between 15 and 30 dBHL. 

Results of the independent *t*-test indicated that there are no significant differences between the two lists (*p* > 0.05) for all presentation hearing levels. Also, the repeated measures analysis results with a Greenhouse–Geisser correction showed no significant interaction on the repeated measures in terms of WRSs for all the conditions: dBHL*List (*p* = 0.498), dBHL*Age (*p* = 0.538), dBHL*Gender (*p* = 0.754), dBHL*List*Age (*p* = 0.858), dBHL*List*Gender (*p* = 0.895), dBHL*Age*Gender (*p* = 0.687), dBHL*List**Age*Gender (*p* = 0.973). 

## 4. Discussion

The results from this study indicate that materials for a WRS test for young children are developed. The test materials have high face validity and are applicable for children as young as three years old. The test satisfies the essential components needed for a WRS test. The aforementioned means that the present test retains the most common features of the large number of WRS tests available in different languages. Such features are the type of speech material, the response format, item scored, phonemic balance, and list equivalency. 

Currently, there is no available information regarding slopes of PI functions for any other Greek picture-pointing test. Therefore, a direct comparison of PI functions is not possible. However, results of an open-set WRS test for Greek-speaking school-aged children 3–6 years revealed an average slope per decibel of 5.08% in the first and 5.24% in the second list in the rising section of the function between 10 and 25 dBHL [4]. Also, PI functions from another open-set WRS test for Greek-speaking individuals over the age of 12 years presented an average slope of 3.37% for the first, 3.35% for the second, 3.31% for the third, and 3.36% for the fourth list per decibel in the rapidly rising portion of the function between 10 and 30 dBHL [18]. PI functions from analogous closed-set tests in other languages revealed comparable results. An English test by Chermak et al. (1988), the Word Intelligibility by Picture Identification Test, reported an average slope per decibel of 5.65%. Another English test, the Northwestern University Children’s Perception of Speech Test, which is presented at a level of 30 dBHL over SRT, reported slopes ranging as high as 3%/dB [9]. A picture-pointing test in the Spanish language [26] presented an increase in recognition ability per decibel of 1.21% (List 1), 1.24% (List 2), and 1.02% (List 3). These values are lower than those from our PI functions because they were calculated based on SRT thresholds, not pure tone thresholds. Thus, some similarity is seen in the slopes of PI functions for both open-set and closed-set tests in Greek and other languages. 

The present test consists of two phonemically balanced 50-word lists with low-redundancy bisyllabic words, with each list containing 227 phonemes. As the speech audiometry materials increase in homogeneity, the given speech audiometry test results become more precise [27]. 

In the present study, we preferred to test the children with full lists (50 words). However, to shorten the testing time, because young children have difficulty maintaining their attention, many clinicians prefer to test with half lists (25 words). An important next step would be to construct four 25-word lists from the above 50-word lists with an effort to maintain the construction criteria and evaluate their equivalency. However, the literature suggests that the variability of a WRS increases as the number of test items decreases and that 50 words are necessary to obtain reliable and valid results [28,29].

The test uses a closed-set response format, with a picture identification task providing a 16.67% chance score more accessible than an open-set format that allows no chance of correct responses through guessing. The closed-set task of this test requires a limited number of comparisons among the six response alternatives. In contrast, an open-set task forces the child to compare the stimulus item to all possible words in lexical memory. In the same line was the study of Clopper et al. [16] that examined the effects of open and closed-set task demands on spoken word recognition. Specifically, Clopper et al. [16] concluded that there is a significant difference in performance between closed-set and open-set word recognition tests and that task difficulty is an important variable to consider when controlling lexical competition. Also, a study [30] reinforces a closed-set domain when assessing speech perception in children. Additionally, the closed-set picture-pointing task can be administered to older children with different deficits, such as severe speech problems or lower cognitive development, impacting scoring accuracy on an open-set test. On the other hand, if the child can cooperate in an open-set format, this test could also be performed as an open-set task. Open-set testing has higher face validity since it is more representative of real-world listening performance. 

The WRSs from the present study were acquired through pre-recorded stimuli. However, most audiologists use monitored live voice (MLV) presentation for WRS testing since it allows the audiologist greater flexibility during the evaluation, especially with young children [31]. Although it is easier for younger children to perform the test with MLV presentation since they cannot maintain consistent attention with pre-recorded stimuli, pre-recorded material is recommended over MLV because of the reduced variability offered by recorded test materials [27]. Moreover, a limitation that needs to be referred to is that the sample should not be considered representative of the population to generalize the results. Future studies should take that into account before their design.

## 5. Conclusions

In conclusion, this study has succeeded in developing a closed-set WRS test for young speakers of Modern Greek. The process included several steps in developing this test, including selecting speech material, digital recordings of speech stimuli, creating and validating digitally illustrated pictures representing the stimuli in the word lists, and establishing list equivalency. This study contributed to establishing a closed-set WRS test in the Greek language that can be valuable in assessing children’s speech perception skills. This test can be of value in assessing speech perception skills in preschool children and older children or adults with limited or no Greek proficiency.

Furthermore, the test could have various applications, including diagnosis, research, and (re)habilitation. Moreover, this study can be a guideline for developing speech audiometry materials in other languages. Also, the results from Greek parts of speech development can offer valuable insights with broader implications for understanding language acquisition and development across various languages since the fundamental principles of language acquisition tend to be universal. This comparative approach can enhance our understanding of language development and support the development of more inclusive and globally applicable strategies for speech-language pathology and early intervention programs.

Regarding future direction, further research with a broad spectrum of children, those whose hearing is within the normal range, those with varying types and degrees of hearing impairment, and those with different developmental abilities is required to establish further validity and reliability of the lists. Moreover, an interesting future investigation would be to compare WRSs for significant differences between a closed and an open-set test format.

## Figures and Tables

**Table 1:** Mean percent scores (%) for the Modern Greek language parts of speech used by children 3–6 years old (n = 300).

Parts of Speech	Frequency of Occurrence
Article	11.98%
Noun	22.65%
Adjective	3.00%
Pronoun	12.64%
Verb	22.25%
Participle	0.16%
Adverb	10.34%
Preposition	4.69%
Conjunction	10.84%
Interjection	0.27%
Particle	1.17%

**Table 2:** Frequency of occurrence of phonemes in Modern Greek spoken language and frequency of preschool lists 1 and 2 (IPA: International Phonetic Alphabet).

	Phonemes IPA Symbol	Frequency Spoken Language	List 1 Frequency (%)	List 2 Frequency (%)
1	a	12.26	11.89	11.45
2	ε	10.40	8.81	8.81
3	i	14.25	13.22	13.22
4	o	9.49	8.37	8.37
5	u	2.50	1.76	2.20
6	ʎ	4.18	4.85	5.29
7	θ	1.11	0.44	0.88
8	ð	2.04	1.32	1.32
9	b	0.26	0.44	0.44
10	d	0.54	1.32	0.88
11	ts	0.11	0.44	0.44
12	dz	0.02	0.44	0.44
13	p	4.36	4.41	5.29
14	m	3.69	3.52	3.52
15	f	1.28	2.20	2.64
16	v	0.88	1.32	1.76
17	t	7.54	6.61	6.61
18	z	0.54	0.44	0.88
19	s	7.68	8.81	7.49
20	n	6.17	4.85	4.85
21	g	0.12	0.44	0.44
22	l	2.77	3.52	3.08
23	k	2.62	3.08	1.76
24	𝒳	0.60	1.32	1.76
25	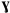	0.74	1.32	1.32
26	c	1.79	1.76	0.88
27	ç	0.88	1.32	2.20
28	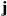	0.98	0.88	0.44
29	ʎ	0.11	0.44	0.88
30	ɲ	0.10	0.44	0.44
Total		100.00	100.00	100.00

**Table 3:** The two bisyllabic word lists for use in preschool WRS testing. (IPA: International Phonetic Alphabet).

List 1				List 2			
Stress		Stress		Stress		Stress	
1st syllable		2nd syllable		1st syllable		2nd syllable	
IPA	Modern Greek	IPA	Modern Greek	IPA	Modern Greek	IPA	Modern Greek
amos	άμμος	avγo	αβγό	ema	αίμα	arɲa	αρνιά
velos	βέλος	afti	αυτί	γadi	γάντι	avli	αυλή
je’ros	γέρος	kafes	καφές	ðiχti	δίχτυ	vuno	βουνό
ðedro	δέντρο	γati	γατί	ðodi	δόντι	vroçi	βροχή
kotes	κότες	garaz	γκαράζ	ðoro	δώρο	γries	γριές
kuɲes	κούνιες	jaʎa	γυαλιά	zugla	ζούγκλα	eʎes	ελιές
kupes	κούπες	evro	ευρώ	zones	ζώνες	pani	πανί
mati	μάτι	ceɾi	κερί	terma	τέρμα	karfi	καρφί
meli	μέλι	kupi	κουπί	tiγris	τίγρης	θeos	θεός
milo	μήλο	kuti	κουτί	nifes	νύφες	keftes	κεφτές
botes	μπότες	laγos	λαγός	θiki	θήκη	kubi	κουμπί
nani	νάνοι	lemos	λαιμός	mites	μύτες	krasi	κρασί
naftes	ναύτες	mali	μαλλί	niçi	νύχι	lefta	λεφτά
pana	πάνα	nona	νονά	niχta	νύχτα	majo	μαγιό
limni	λίμνη	scini	σκοινί	porta	πόρτα	moro	μωρό
roða	ρόδα	peði	παιδί	pites	πίτες	nero	νερό
teda	τέντα	kapnos	καπνός	pçano	πιάνο	palto	παλτό
dzaci	τζάκι	scepi	σκεπή	sela	σέλα	papas	παπάς
tiχos	τοίχος	spaθi	σπαθί	dzami	τζάμι	papi	παπί
topi	τόπι	tapsi	ταψί	tuvlo	τούβλο	puʎa	πουλιά
tsada	τσάντα	tiri	τυρί	treno	τρένο	nisi	νησί
çeri	χέρι	fili	φιλί	tsepi	τσέπη	taksi	ταξί
çoni	χιόνι	ftero	φτερό	fetes	φέτες	fotça	φωτιά
χtenes	χτένες	χarti	χαρτί	çines	χήνες	χali	χαλί
psari	ψάρι	çimos	χυμός	χoma	χώμα	psomi	ψωμί

## Data Availability

Available after request to the corresponding author.
